# Emergence of Oropouche Virus, Venezuela, 2025

**DOI:** 10.3201/eid3209.250678

**Published:** 2026-09

**Authors:** Pierina D’Angelo, Lieska Rodríguez, Víctor Alarcón, Yoneira Sulbarán, Norca García, Giulianna Antonelli, Marwan Aguilar, Martha Bravo, Zoila C. Moros, Carmen L. Loureiro, Ferdinando Liprandi, José L. Zambrano, Rossana C. Jaspe, Flor H. Pujol

**Affiliations:** Instituto Nacional de Higiene “Rafael Rangel,” Caracas, Venezuela (P. D’Angelo, L. Rodríguez, V. Alarcón, N. García, G. Antonelli, M. Aguilar, M. Bravo); Instituto Venezolano de Investigaciones Científicas, Caracas (Y. Sulbarán, Z.C. Moros, C.L. Loureiro, F. Liprandi, J.L. Zambrano, R.C. Jaspe, F.H. Pujol)

**Keywords:** Oropouche virus, arbovirus, vector-borne infections, zoonoses, genotype, outbreak, viruses, Venezuela

## Abstract

We describe 5 cases of human Oropouche virus infection detected in rural Venezuela in 2025. All isolates belonged to the OROVBr_2015–2024_ reassortant lineage linked to the 2024–2025 epidemic. Our results emphasize ongoing transmission, which highlights the need for strengthened surveillance and laboratory capacity in South America.

Oropouche virus (OROV) is an arthropodborne virus and member of the family Peribunyaviridae, genus *Orthobunyavirus*; the species is *Orthobunyavirus oropoucheense*. The genome consists of 3 segments of a linear negative-stranded RNA: small, medium, and large (*1*). Several outbreaks of OROV infection have occurred in the Amazon region since 1960. The largest OROV outbreak has been ongoing since 2022 (*1*). By February 2025, a total of 9 countries in the Americas reported autochthonous cases of OROV. Meanwhile Canada, Cayman Islands, the United States, Germany, Italy, and Spain also reported 30 imported cases, mostly in travelers returning from Cuba (*2*).

The National Institute of Hygiene Rafael Rangel (Caracas, Venezuela) has been detecting OROV by using reverse transcription PCR since 2023. Beginning in 2024, a total of 25% of the samples negative for dengue, chikungunya, and Zika viruses were tested for Mayaro and Oropouche viruses by quantitative reverse transcription PCR (qRT-PCR) (*3*). During the first trimester of 2025, five samples tested positive for OROV by qRT-PCR in 2 states of Venezuela, Miranda and Portuguesa (Figure 1). After the first case of OROV was detected in January 2025, all the samples negative for the 3 other arboviruses from January–March 2025 were retrospectively tested for Mayaro virus and OROV, which were all negative. This study was approved by the Bioethical Committee of Instituto Venezolano de Investigaciones Científicas (protocol no. CBioEtIVIC-2022–001): written informed consent was obtained in OROV-positive human cases. We described the clinical findings of the 5 patients in accordance with the Pan American Health Organization guidelines (Appendix Table 1) (*2*). We observed no neurologic compromise in those cases. For the first case, the patient had a possible relapse 3 weeks later, although qRT-PCR was negative. Recurrence seems to be a frequent phenomenon in OROV infection (*4*).

**Figure 1:**
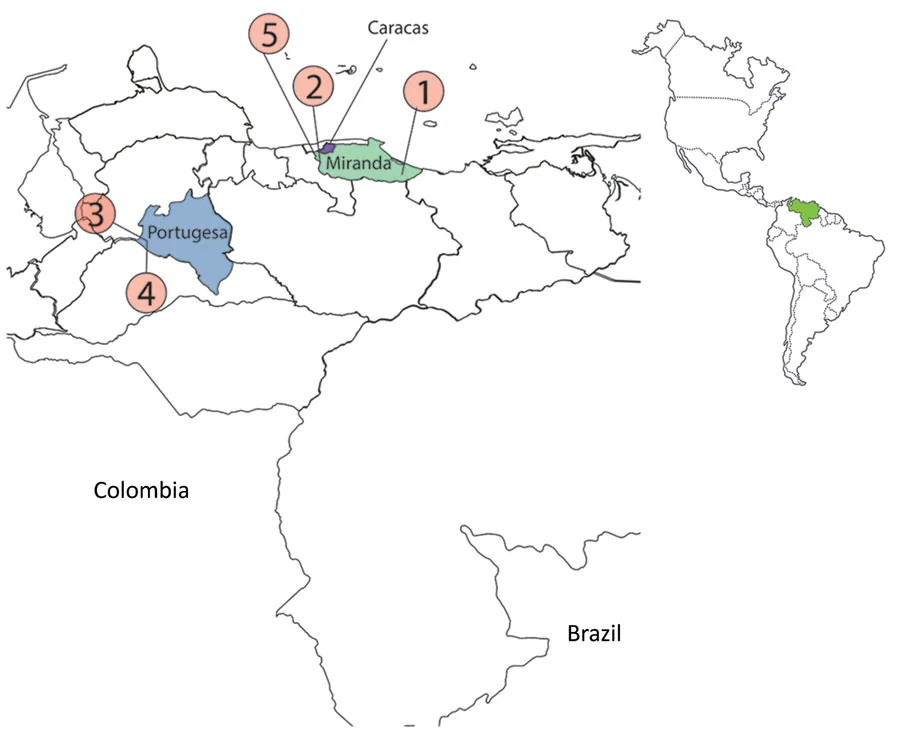
Locations of Oropouche virus cases detected in Venezuela, 2025. The numbers represent the cases detected. The cases were detected in the Portugesa and Miranda states. The first detected Oropouche virus case was from January 27, 2025, although official notification was performed in March 2025, when sequences were available. Inset shows location of Venezuela in the Americas.

We collected serum samples 2 or 3 days after the appearance of acute febrile illness. We performed genome sequencing on an Illumina iSeq platform (Illumina, https://www.illumina.com) by using a COVIDSeq library and primers specific for the OROV genome (Appendix Table 2) (*2*). We assembled genome sequences by using both Genome Detective (https://www.genomedetective.com) and INSaFLU-TELEVIR bioinformatic tools (https://insaflu.insa.pt).

We deposited complete genome sequences into the GISAID database (accession nos. EPI_ISL_19859479–81), and in GenBank (GenBank accession nos. PX494099–101, PX495048–50 and PX495206–8). We aligned sequences by using MAFFT (*5*) and performed phylogenetic analysis by using IQTree with 1000 bootstrap replicas (Appendix) (*6*).

Our phylogenetic analysis of 3 complete viral genome segments revealed that the isolates from Venezuela grouped in the OROV_BR2015–2024_ clade, a dominant lineage associated with current outbreaks (*1*). The sequences from Venezuela were related to OROV isolates from Cuba. However, the small segment sequences were also closely related to sequences from Brazil (Figure 2). A large outbreak of OROV occurred in Cuba in 2024, with 626 confirmed cases, and was still ongoing in 2025 (*2*). Since late 2022, an unprecedented epidemic of OROV infection is still ongoing in Brazil (*1*,*2*,*7*). However, no history of travel outside the country was reported for the cases in Venezuela.

**Figure 2:**
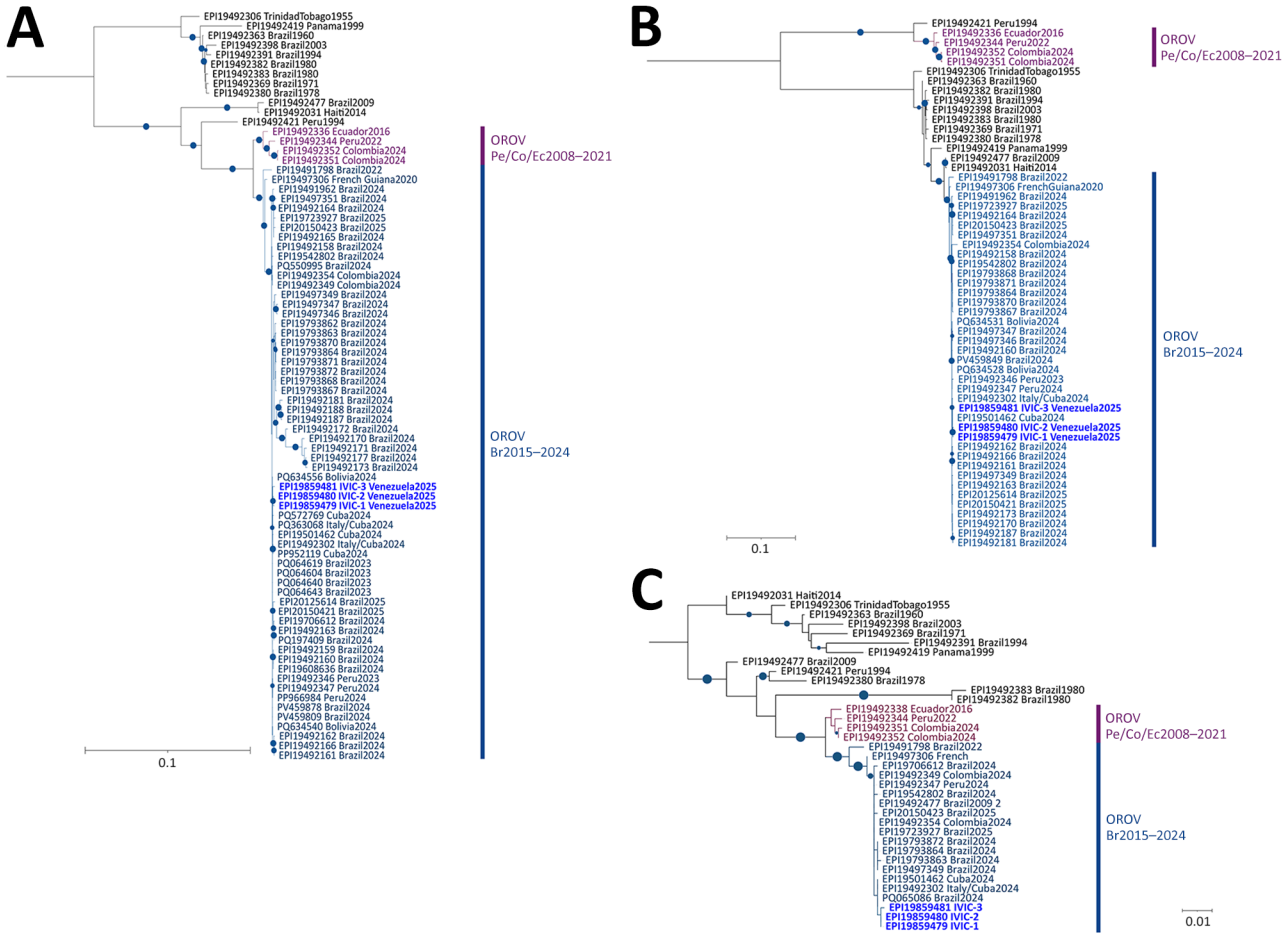
Phylogenetic analysis of OROV cases in Venezuela, 2025. Maximum-likelihood phylogenetic analysis for each genomic segment of the OROV genome: A) large genome segment sequences; B) medium genome segment sequences; C) small genome segment sequences. The isolates are named according to their accession number in GenBank or GISAID (https://www.gisaid.org), including the country and year of collection of each sample. Blue indicates the OROV_BR2015–2024_ clade and purple the OROV_Pe/Co/Ec2008–2021_ clade. Bold indicates the sequences of the Venezuela isolates. The tree scale indicates the average number of nucleotide substitutions per site. Blue circles indicate bootstrap values >80. OROV, Oropouche virus.

A close relative of OROV, Madre de Dios Virus, was isolated from a monkey (*Cebus olivaceus*) during an epizootic outbreak in the Eastern region of Venezuela in 2010 (*8*). Animal reservoirs, such as nonhuman primates and sloths, cannot be excluded in other rural regions of Venezuela and might be involved in a spillover OROV event to humans (*9*). However, the relatedness of the sequences to the OROV_BR2015–2024_ clade suggests an introduction from the countries with OROV outbreaks. The 2 cases from Portuguesa state were from close localities, in contrast with the cases from Miranda state. No secondary cases were found among febrile persons in the localities where the OROV positive cases were found. However, secondary asymptomatic cases (*10*) cannot be ruled out. Epidemiologic surveillance of OROV infection is challenging because of the dengue virus epidemic in Venezuela. Some OROV cases might be underreported because they are misdiagnosed as dengue virus, or because they were asymptomatic (*10*). Two additional OROV infections were reported in April 2025 in Portuguesa state.

In conclusion, we detected OROV in Venezuela in 2025. Although underreporting of OROV cases cannot be ruled out, the epidemiologic pattern of this viral infection in Venezuela does not appear to be as pervasive compared with other countries such as Brazil and Cuba. We believe continued surveillance in the region is necessary to reduce outbreak risk. 

Appendix Additional information about emergence of Oropouche virus, Venezuela, 2025
