# Supplementary material for: Emergence of Oropouche Virus, Venezuela, 2025
Source: Emerg Infect Dis. 2026 Sep;32(9):1523–5. doi: 10.3201/eid3209.250678 (PMC13555709; doi:10.3201/eid3209.250678)
Supplement: Supplementary file 1 [file 25-0678-Techapp-s1.pdf]

Article DOI: https://doi.org/10.3201/eid3209.250678

EID cannot ensure accessibility for supplementary materials supplied by authors. Readers who have difficulty accessing supplementary content should contact the authors for assistance.

Emergence of Oropouche Virus, Venezuela, 2025

Appendix

Complete genome sequencing was performed by Next-Generation Sequencing. Libraries were prepared using the Illumina COVIDSeq Assay (96 Samples) (RUO Version, Document # 1000000126053 v05), replacing SARS-CoV-2 specific primers with primers targeting the OROV genome (1), and using IDT for Illumina-PCR Indexes set 1. The libraries were pooled, quantified (Qubit DNA HS, Thermo Scientific, Waltham, MA, USA), and their quality assessed via a Bio-Fragment Analyzer (Qsep1-Lite, BiOptic, New Taipei City, People's Republic of China) before sequencing, which was performed with 10% PhiX control v3, using an iSeq 100 platform and a 300 cycle V2 kit with paired-end reads.

For the phylogenetic analysis, a Blast analysis was performed for each segment of each Venezuelan isolate, both in GenBank and GISAID databases, and the first five non redundant sequences (not selected already for another isolate) displaying the highest identity with the Venezuelan sequences were included in the tree. Oropouche virus sequences from other South American countries available in either database, spanning each decade before this study, were also included. For Brazil, the country reporting most of the sequences available, a sequence was selected for each semester of collection over the past 6 years. Reference sequences were also used as outgroup. Identical sequences from the same country were removed in the corresponding tree. Alignment was performed using MAFFT (*5*). The 5′ and 3′ ends of the longest sequences were manually trimmed. Maximum likelihood analysis was performed using IQ-TREE (6). The best-fit substitution model was assessed for each genomic segment: TN+F+I+G4 for the L and M segments, and HKY+F+I for the S segment. A total of 1,000 replicates was performed for each tree.

Appendix Table 1. Clinical description of the five Oropouche cases detected in Venezuela, January-March 2025

Case	IVIC-1	IVIC-2	IVIC-3	IVIC-4	IVIC-5
Living address	Los Teques Miranda State	El Clavo Miranda State	Boconoito Portuguesa State	Boconoito Portuguesa State	La Dolorita Miranda State
Sex and age	Male, 59 y	Male, 12 y	Female, 20 y	Male, 8 y	Male, 8 y
Symptoms	Fever, headache, retroocular pain, myalgia, arthralgia and fatigue	Fever, headache, retroocular pain, rash, myalgia and arthralgia	Fever, headache, retroocular pain, rash, myalgia, arthralgia, abdominal pain, vomiting and gingivorrhea	Fever, headache, retroocular pain, rash, myalgia and arthralgia, chill, abdominal pain and odynophagia	Fever, anorexia, fatigue, cough
Date of onset of symptoms	January 27, 2025 Relapse on February 15*	February 2, 2025	February 17, 2025	March 14, 2025	March 17, 2025
Date of collection of serum	January 30, 2025	February 5, 2025	February 19, 2025	March 17, 2025	March 19, 2025
Ct (qRT-PCR)	25	32	25	38	24
History of travel outside Venezuela	No	No	No	No	No

*qRT-PCR for the sample collected on February 19 was negative at this time but was also negative for the other arboviruses tested: Mayaro, Zika, dengue and Chikungunya.

Appendix Table 2. Sequencing of the Oropouche viral genome*

Sample ID	GISAID ID/GenBank AN	Ct value	Gene	Length pb	Reads	Depth of coverage	Coverage (%)
IVIC-1	19859479/	25	S	720	146826	21128.4	95.5%
	PX494099-		M	4353	35161	889.6	99.3%
	PX494101		L	6733	45050	699.3	98.3%
IVIC-2	19859480/	32	S	720	201378	28720.5	95.5%
	PX495048-		M	4351	33482	846.3	99.2%
	PX495050		L	6732	51833	804.2	98.3%
IVIC-3	19859481/	25	S	719	213625	30369.5	95.4%
	PX495206-		M	4354	37270	937.3	99.3%
	PX495208		L	6737	60048	932.4	98.4%
IVIC-4	NA	38	M	407	39454	9798.3	9.30%
IVIC-5	NA	24	NA†				

*AN, accession number; NA, not available

†No reads were obtained for this sample, probably due to RNA degradation during transportation from one laboratory to another.
